# Association of High-Deductible Health Plan Enrollment With Spending on and Use of Lenalidomide Therapy Among Commercially Insured Patients With Multiple Myeloma

**DOI:** 10.1001/jamanetworkopen.2022.15720

**Published:** 2022-06-07

**Authors:** Shelley A. Jazowski, Lauren Wilson, Stacie B. Dusetzina, S. Yousuf Zafar, Leah L. Zullig

**Affiliations:** 1 Department of Health Policy and Management, UNC (University of North Carolina at Chapel Hill) Gillings School of Global Public Health; 2 Department of Health Policy, Vanderbilt University School of Medicine, Nashville, Tennessee; 3 Department of Population Health Sciences, Duke University School of Medicine, Durham, North Carolina; 4 Vanderbilt-Ingram Cancer Center, Nashville, Tennessee; 5 Division of Medical Oncology, Department of Medicine, Duke University School of Medicine, Durham, North Carolina; 6 Sanford School of Public Policy, Duke University, Durham, North Carolina; 7 Center of Innovation to Accelerate Discovery and Practice Transformation, Durham Veterans Affairs Health Care System, Durham, North Carolina

## Abstract

**Importance:**

High-deductible health plans (HDHPs) require high upfront cost-sharing, which has been associated with suboptimal anticancer medication uptake and adherence. Whether HDHP enrollment has limited the affordability and use of lenalidomide therapy among commercially insured patients with multiple myeloma is unknown.

**Objective:**

To assess the association of HDHP enrollment with out-of-pocket spending on and adherence to lenalidomide therapy.

**Design, Setting, and Participants:**

In this cohort study, data were obtained from the IBM MarketScan Commercial Claims and Encounters Database for adults aged 18 to 64 years with multiple myeloma who newly initiated lenalidomide therapy between April 1, 2013, and June 30, 2017. Quantile regression and modified Poisson regression evaluated out-of-pocket spending, and group-based trajectory models and multinomial logistic regression examined patterns of and factors associated with adherence. Analyses were conducted from April to August 2020.

**Exposures:**

High-deductible health plan enrollment in the 3 months before and 6 months after initiation of lenalidomide therapy.

**Main Outcomes and Measures:**

Distribution of out-of-pocket spending, the probability of paying more than $100 for the first and any lenalidomide prescription fill, and monthly lenalidomide therapy adherence using the proportion of days covered (≥80%).

**Results:**

Of the 3163 commercially insured patients who initiated lenalidomide therapy (median age, 57 years [IQR, 53-60 years for HDHP enrollees and 52-61 years for non-HDHP enrollees]), 328 (10.4%) were enrolled in HDHPs and 1769 (55.9%) were women. Among the highest spenders (95th percentile), HDHP enrollees paid $376 (95% CI, −$28 to $780) and $217 (95% CI, $106-$323) more for their first and any lenalidomide prescription fill, respectively, compared with non-HDHP enrollees in the 6 months after initiation. High-deductible health plan enrollment was also associated with an increased risk of paying more than $100 for the initial (adjusted risk ratio [aRR], 1.30 [95% CI, 1.13-1.50]) and any (aRR, 1.26 [95% CI, 1.12-1.42]) lenalidomide prescription fill. Three adherence trajectory groups were identified: those with high adherence (n = 1273), late nonadherence (n = 1084), and early nonadherence (n = 805). High-deductible health plan enrollment was not associated with adherence group assignment.

**Conclusions and Relevance:**

In this cohort study, HDHP enrollment was associated with higher out-of-pocket spending per lenalidomide prescription fill; however, no statistically significant differences in adherence patterns between HDHP and non-HDHP enrollees were observed. Patient (eg, perceptions of treatment benefits), payer (eg, out-of-pocket maximums), and clinician (eg, counseling patients on disease severity) factors may have limited the potential for nonadherence among commercially insured patients who initiated lenalidomide therapy.

## Key Points

**Question:**

How does out-of-pocket spending on and the use of lenalidomide therapy differ between commercially insured patients enrolled in high-deductible health plans (HDHPs) and non-HDHPs?

**Findings:**

In this cohort study of 3163 adults who initiated lenalidomide therapy between 2013 and 2017, HDHP enrollment was associated with a greater likelihood of paying more than $100 per lenalidomide prescription fill compared with non-HDHP enrollment. However, no meaningful differences in patterns of lenalidomide therapy adherence were observed by enrollment status.

**Meaning:**

Despite higher observed out-of-pocket spending among commercially insured HDHP enrollees, patterns of lenalidomide therapy adherence were similar between HDHP and non-HDHP enrollees.

## Teaser

This cohort study assesses the association of high-deductible health plan enrollment with out-of-pocket spending on and adherence to lenalidomide therapy among commercially insured patients with multiple myeloma.

## Introduction

High-deductible health plans (HDHPs) are one of the preferred forms of commercial insurance coverage in the US.^1,2,3^ In 2019, more than 56% of large employers (those with ≥200 employees) offered and approximately half of their employees were enrolled in HDHPs.^3,4,5^ High-deductible health plans require substantial upfront out-of-pocket costs, with mean annual deductibles for large employer-sponsored plans ranging from approximately $2500 for individual coverage to $5300 for family coverage.^3^ Although HDHPs are designed to curb the overuse of health care services and spending on low-value care,^6,7,8,9^ high cost-sharing could exacerbate the financial burden of patients with cancer^6,9,10^ and limit access to necessary care.^9,10^

Research has demonstrated that HDHP enrollment is associated with the suboptimal use of anticancer therapy.^1,7,10,11^ For example, HDHP enrollees often delay chemotherapy initiation for 5.1 to 8.7 months^1,7^ and report both cost-related nonadherence (eg, taking less medication than prescribed) and the use of cost-coping strategies (eg, requesting lower-cost and/or alternative treatments).^10^ Although these studies establish a foundation of research exploring the role of HDHP enrollment on anticancer therapy use more broadly, questions regarding whether HDHPs affect the affordability of and adherence to novel, orally administered antimyeloma medications remain.

During the past decade, the cost of orally administered antimyeloma therapy has dramatically risen, with monthly treatment costs exceeding $10 000.^12,13,14^ High treatment costs are especially concerning for patients who often receive therapy indefinitely,^15,16^ are switched to another high-cost treatment at relapse,^13,14^ and/or are prescribed regimens consisting of 3 or more medications.^12,13,16,17^ Lenalidomide is used at different points throughout the course of treatment, including initial therapy,^13,17,18^ maintenance therapy after a stem cell transplant,^13,17,18^ and as part of multidrug regimens to treat patients who have relapsed.^17,19^ Given both the widespread use and high cost (>$760 per pill)^13,14,20^ of lenalidomide therapy, our objectives were to assess the association of HDHP enrollment with out-of-pocket spending on and adherence to lenalidomide therapy among commercially insured patients with multiple myeloma.

## Methods

### Data Source and Study Population

In this cohort study, we used the IBM MarketScan Commercial Claims and Encounters Database to identify adults with multiple myeloma (*International Classification of Diseases, Ninth Revision*, code 203.00 and *International Statistical Classification of Diseases and Related Health Problems, Tenth Revision*, code C90.0) who initiated lenalidomide therapy between April 1, 2013, and June 30, 2017. Eligible patients were aged 18 to 64 years at initiation of lenalidomide therapy, had at least 12 months of continuous health plan coverage in a year with a claim for multiple myeloma, had continuous health plan coverage at least 3 months before and 6 months after their initial lenalidomide prescription fill, and had no evidence of prior treatment with lenalidomide in the 3-month baseline period. Race and ethnicity data were not collected because these data were not available in the data set. This study was deemed exempt from approval and informed consent by the Duke Health Institutional Review Board and conformed to the Strengthening the Reporting of Observational Studies in Epidemiology (STROBE) reporting guideline.

### Outcomes

The primary outcome was out-of-pocket spending on the initial and any lenalidomide prescription fill in the 6 months after initiation. Out-of-pocket costs (sum of deductible, coinsurance, and copayments) were standardized to a 28-day supply and inflation adjusted to 2017 dollars using the medical component of the Consumer Price Index.^15,21^ We separately assessed the distribution of out-of-pocket spending and the probability of paying more than $100 per lenalidomide prescription fill. Consistent with prior studies, we selected more than $100 because the rate of anticancer treatment delays and prescription abandonment increases significantly at this spending level.^15,22^

Adherence to lenalidomide therapy was also a primary outcome. We measured adherence using the proportion of days covered, which represents the days a patient had lenalidomide therapy available divided by the days in the study period (each month during a 6-month period).^23^ In accordance with prior research and Pharmacy Quality Alliance thresholds, patients were considered adherent to lenalidomide therapy if the monthly proportion of days covered was at least 80%.^15,23,24^

### Exposure and Covariates

The key independent variable was enrollment in an HDHP. Patients were categorized as HDHP enrollees if they were covered by an HDHP throughout the entirety of the study period (ie, 3 months before and 6 months after lenalidomide therapy initiation); otherwise, they were classified as a non-HDHP enrollee (ie, those with comprehensive coverage or enrolled in exclusive provider organization plans, health maintenance organization plans, point of service plans, point of service plans with capitation, preferred provider organization plans, or consumer-driven health plans). Covariates included patient demographics (age at initiation of lenalidomide therapy and sex), region, quarter and year of lenalidomide therapy initiation, comorbidities (measured in the 3 months before the initial lenalidomide prescription fill using the Klabunde modification of the Charlson Comorbidity Index),^25^ and number of unique prescription medications in the 3-month baseline period.

### Statistical Analysis

We used quantile regression to evaluate the distribution of out-of-pocket spending at the 25th, 50th, 75th, 90th, and 95th percentiles for the initial and any lenalidomide prescription fill and modified Poisson regression with robust error variance^26^ to estimate the probability of paying more than $100 out of pocket for the initial and any lenalidomide prescription fill.

Following previous studies, we evaluated adherence with group-based trajectory models, which simultaneously estimate group assignment probabilities and longitudinal adherence trajectories.^27,28,29^ Specifically, 6 monthly adherence indicators were assessed using logistic regression for group-specific models, with time defined as the months after lenalidomide therapy initiation.^27,28,29^ We estimated models consisting of 2 to 5 adherence groups and used third-order polynomials to model adherence over time.^28,29,30^ We compared fit across models and determined the optimal number of adherence groups with bayesian information criteria^27,28,29,30,31^ and estimated group proportions (≥5% of the study population was assigned to each group).^27,30,31,32^ After determining the most appropriate group-based trajectory model, we used multinomial logistic regression to assess the association between HDHP enrollment and adherence group assignment.^30,31^

For all models, we estimated unadjusted and adjusted outcomes with SAS, version 9.4 (SAS Institute, Inc). Analyses were conducted from April to August 2020. Statistical tests were 2 sided, and *P* < .05 denoted statistical significance.

### Sensitivity Analysis

We conducted several sensitivity analyses. First, the time of year in which lenalidomide therapy was initiated may have influenced both out-of-pocket costs and adherence for HDHP enrollees (eg, initiation in the beginning of the year is associated with higher spending and potentially suboptimal adherence for enrollees who have not yet met their deductible and out-of-pocket maximum).^10,33^ Therefore, we included the interaction between quarter of initiation and HDHP enrollment in all models. Second, to account for high antimyeloma treatment costs and variation in out-of-pocket maximums across health plans, we used modified Poisson regression with robust error variance^26^ to estimate the probability of paying $0 for the first and any lenalidomide prescription fill.

## Results

### Study Population Characteristics

**Figure 1.**
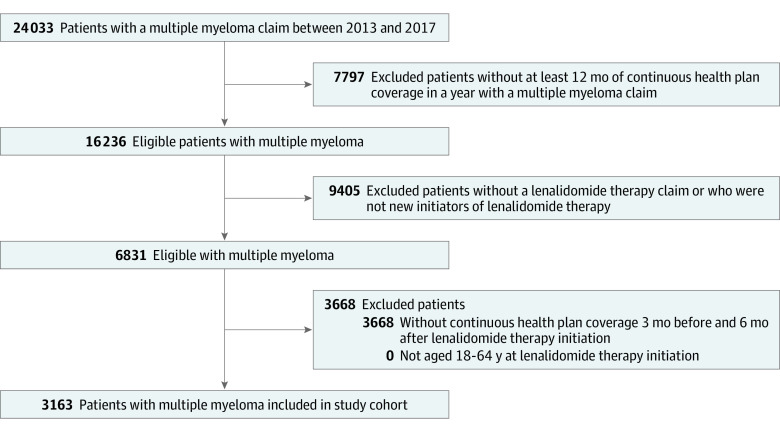
Study Flow Diagram

Among the 3163 commercially insured adults who initiated lenalidomide therapy between April 1, 2013, and June 30, 2017 (1769 women [55.9%] and 1394 men [44.1%]) (Figure 1), 328 (10.4%) were enrolled in HDHPs and 2835 (89.6%) were covered by non-HDHPs (Table 1). Baseline demographic and clinical characteristics were similar across enrollees: most HDHP and non-HDHP enrollees were women (190 [57.9%] vs 1579 [55.7%], respectively), and the median age at initiation was 57 years for both HDHP (IQR, 53-60 years) and non-HDHP (IQR, 52-61 years) enrollees. However, a lower percentage of HDHP enrollees initiated lenalidomide therapy in 2013 (45 [13.7%] vs 639 [22.5%]) and resided in the northeastern US (34 [10.4%] vs 580 [20.5%]) compared with non-HDHP enrollees.

**Table 1.** Baseline Characteristics of HDHP and Non-HDHP Enrollees

Characteristic	Enrollee group^a^		*P* value
	HDHP (n = 328)	Non-HDHP (n = 2835)^b^	
Age at initiation, median (IQR), y	57 (53.0-60.0)	57 (52.0-61.0)	.91
Sex			
Women	190 (57.9)	1579 (55.7)	.44
Men	138 (42.1)	1256 (44.3)	
US region			
Northeast	34 (10.4)	580 (20.5)	<.001
North central	83 (25.3)	570 (20.1)	
South	177 (54.0)	1239 (43.7)	
West	33 (10.1)	399 (14.1)	
Unknown	1 (0.3)	47 (1.7)	
Year of initiation			
2013	45 (13.7)	639 (22.5)	<.001
2014	66 (20.1)	662 (23.3)	
2015	72 (21.9)	603 (21.3)	
2016	93 (28.3)	632 (22.3)	
2017	52 (15.9)	299 (10.5)	
Quarter of initiation			
First	92 (28.0)	751 (26.5)	.50
Second	112 (34.1)	965 (34.0)	
Third	71 (21.6)	567 (20.0)	
Fourth	53 (16.1)	552 (19.5)	
Comorbidities^c^			
0	230 (70.1)	1965 (69.3)	.94
1	63 (19.2)	568 (20.0)	
≥2	35 (10.7)	302 (10.7)	
Unique prescription medications, median (IQR), No.^c^	7 (4.0-10.0)	7 (4.0-10.0)	.96

Abbreviation: HDHP, high-deductible health plan.

^a^ Data are expressed as number (%) of enrollees unless indicated otherwise. Percentages are rounded and may not total 100.

^b^ Non-HDHP plans included comprehensive coverage, exclusive provider organization plans, health maintenance organization plans, point of service plans, point of service plans with capitation, preferred provider organization plans, and consumer-driven health plans.

^c^ Measured in the 3 months before lenalidomide therapy initiation.

### Distribution of Out-of-Pocket Spending

**Figure 2.**
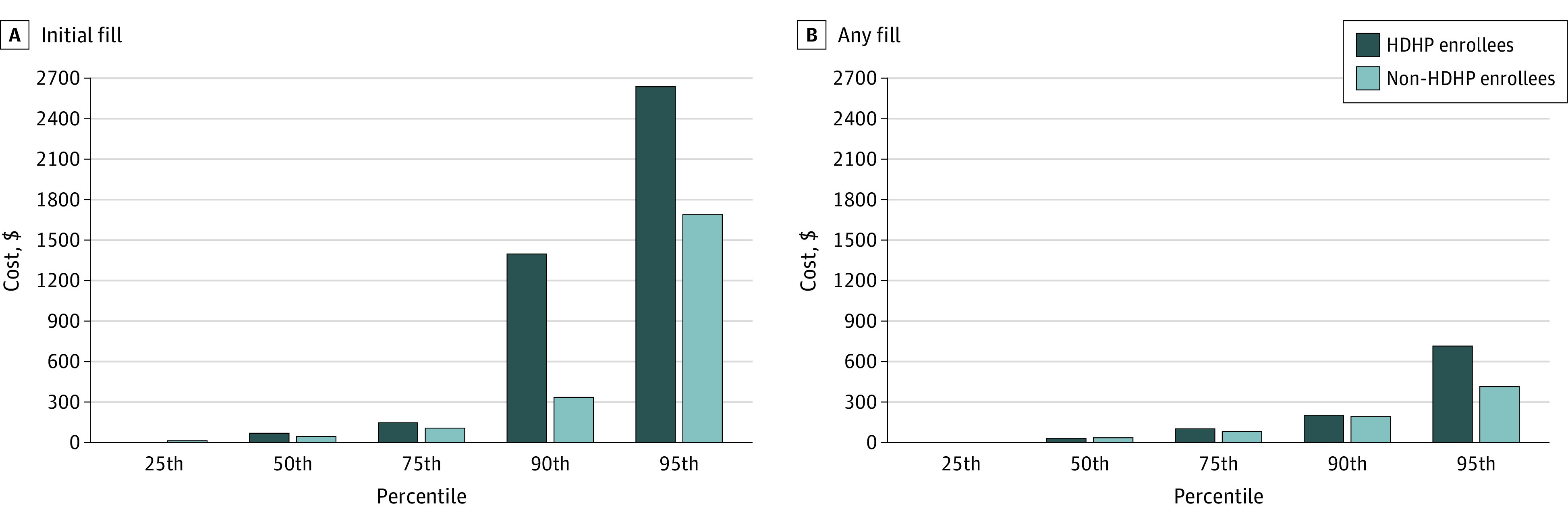
Unadjusted Out-of-Pocket Costs for Lenalidomide Prescription Fills by Spending Quantile Data represent unadjusted spending quantiles for high-deductible health plan (HDHP) and non-HDHP enrollees for the initial and any lenalidomide prescription fill.

In unadjusted analyses, HDHP enrollees paid $1063 (95% CI, $645-$1482) and $946 (95% CI, −$26 to $1918) more for their initial lenalidomide prescription fill at the 90th and 95th percentiles of out-of-pocket spending, respectively, relative to non-HDHP enrollees (Figure 2A). Similar trends in out-of-pocket costs were observed among the highest spenders in adjusted models (differences in spending between HDHP and non-HDHP enrollees were $756 [95% CI, −$859 to −$654] at the 90th percentile and $376 [95% CI, −$780 to $28] at the 95th percentile) (eTable 1 in the Supplement).

When assessing the distribution of unadjusted out-of-pocket costs on any prescription fill of lenalidomide in the 6 months after initiation, HDHP enrollees paid more at the 75th ($21 [95% CI, $13-$29]), 90th ($8 [95% CI, −$24 to $40]), and 95th ($299 [95% CI, $85-$512]) quantiles of spending than non-HDHP enrollees (Figure 2B). Adjusted out-of-pocket spending varied between enrollees (eTable 2 in the Supplement). Specifically, non-HDHP enrollees paid $8 (95% CI, $6-$9) more than HDHP enrollees at the lowest quantile of spending, whereas HDHP enrollees paid $217 (95% CI, $106-$323) more than non-HDHP enrollees at the 95th percentile of spending.

### Probability of Paying More Than $100 Out of Pocket

A larger proportion of HDHP enrollees paid more than $100 for both their first (133 [40.5%] vs 812 [28.6%]) and any (160 [48.8%] vs 1008 [35.5%]) lenalidomide prescription fill vs non-HDHP enrollees (Table 2). Compared with non-HDHP enrollees, HDHP enrollees had a 30% (adjusted risk ratio [aRR], 1.30 [95% CI, 1.13-1.50]) and 26% (aRR, 1.26 [95% CI, 1.12-1.42]) higher probability of paying more than $100 for their initial and any lenalidomide prescription fill, respectively.

**Table 2.** Association Between HDHP Enrollment and Paying More Than $100 for Lenalidomide Prescription Fills

Lenalidomide prescription fill	Enrollees, No. (%)		RR (95% CI)	
	HDHP	Non-HDHP	Unadjusted	Adjusted^a^
Initial fill >$100 out-of-pocket costs	133 (40.5)	812 (28.6)	1.42 (1.23-1.63)	1.30 (1.13-1.50)
Any fill >$100 out-of-pocket costs	160 (48.8)	1008 (35.5)	1.37 (1.22-1.55)	1.26 (1.12-1.42)

Abbreviations: HDHP, high-deductible health plan; RR, risk ratio.

^a^ Adjusted for age at lenalidomide therapy initiation, sex, region, quarter and year of lenalidomide therapy initiation, comorbidities, and unique prescriptions at baseline.

### Adherence

**Figure 3.**
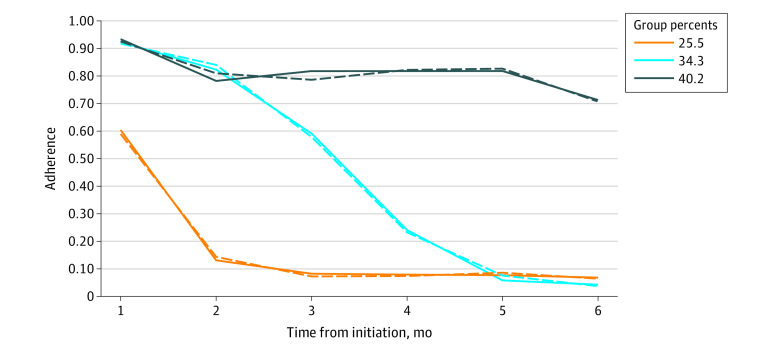
Trajectories of Lenalidomide Therapy Adherence in the 6 Months After Treatment Initiation Trajectories of lenalidomide therapy adherence in the 6 months after initiation were generated using PROC TRAJ in SAS, version 9.4 (SAS Institute, Inc). The observed sample proportion is plotted with solid lines; the estimated trajectory of each group, with dashed lines.

After evaluating model fit (eTable 3 in the Supplement), we identified 3 adherence groups among patients who initiated lenalidomide therapy: those with high adherence (1273 [40.2%]), late nonadherence (1084 [34.3%]), and early nonadherence (805 [25.5%]) (Figure 3). In both unadjusted and adjusted multinomial regression models, HDHP enrollment was not associated with adherence group assignment (eTable 4 in the Supplement).

### Sensitivity Analysis

#### Interaction Between Enrollment and Quarter of Initiation

Unadjusted and adjusted out-of-pocket spending on the initial lenalidomide prescription fill was higher at every quantile of spending for patients who initiated treatment in the first quarter of the year compared with those who started therapy in the fourth quarter (eTable 5 in the Supplement). Among the highest spenders (90th and 95th percentiles), both HDHP and non-HDHP enrollees who started treatment in the first quarter paid 3 to 12 times more than their counterparts who initiated lenalidomide therapy in the fourth quarter. Although unadjusted and adjusted out-of-pocket costs were high for all patients initiating lenalidomide therapy in the first quarter, HDHP enrollees consistently paid 2 to 3 times more than non-HDHP enrollees at the 50th, 75th, and 90th percentiles of spending.

When assessing unadjusted and adjusted out-of-pocket spending on any prescription fill of lenalidomide in the 6 months after initiation, HDHP and non-HDHP enrollees who started treatment in the first and fourth quarters of the year had consistently higher spending at the 50th to 95th percentiles than their counterparts who began therapy in the second and third quarters of the year (eTable 6 in the Supplement). At the 95th percentile of spending, HDHP enrollees who started treatment in the first and fourth quarters paid more than twice as much as non-HDHP enrollees.

For models evaluating the probability of paying more than $100 per lenalidomide prescription fill and the association between enrollment status and adherence group assignment, the interaction between HDHP enrollment and quarter of initiation was not statistically significant.

#### Probability of Paying $0 Out of Pocket

Fewer than one-third of HDHP and non-HDHP enrollees paid $0 for their initial lenalidomide prescription fill, whereas a larger percentage of HDHP enrollees (202 [61.6%] vs 1069 [37.7%]) paid $0 for any lenalidomide prescription fill in the 6 months after initiation (eTable 7 in the Supplement). Compared with non-HDHP enrollees, HDHP enrollees were more likely to pay $0 for their first (aRR, 1.25 [95% CI, 1.04-1.50]) and any (aRR, 1.64 [95% CI, 1.48-1.82]) lenalidomide prescription fill.

## Discussion

In this cohort study comparing out-of-pocket spending on and use of lenalidomide therapy between commercially insured HDHP and non-HDHP enrollees, we observed that HDHP enrollees were more likely to pay more than $100 per prescription fill in the 6 months after initiation. In addition, HDHP enrollees at the 90th and 95th percentiles of out-of-pocket spending often paid more than $2000 for their initial lenalidomide prescription fill. Because HDHP enrollment is projected to increase dramatically during the next decade^1^ and more US residents report less than $2000 in liquid assets,^34^ addressing high out-of-pocket spending is critical. Policy makers and payers should consider transitioning from annual to monthly out-of-pocket maximums^34,35^ and waiving deductibles (as is currently the case for chronic medications such as insulin, statins, and inhaled corticosteroids)^36^ to ensure greater financial security^34^ for and optimal use of high-value anticancer medications by enrollees.

Although HDHP enrollment was associated with high out-of-pocket spending, we did not observe statistically significant differences in high adherence between HDHP and non-HDHP enrollees. Out-of-pocket costs among the lowest spenders (25th and 50th percentiles) were perhaps too small ($1 to $53 per fill) to adversely affect continued use of lenalidomide therapy,^2,33^ whereas the highest spenders (90th and 95th percentiles) may have adopted a variety of strategies to aid with monthly prescription expenses. For example, HDHP and non-HDHP enrollees may have used prescription assistance programs (eg, pharmaceutical manufacturer coupons, copayment support),^15,37^ personal or employer-sponsored financial resources (eg, household income, health spending accounts),^10,38^ or cost-coping strategies (eg, reduced spending on necessities or leisure activities)^39^ to maintain use of lenalidomide therapy. Advocacy organizations and policy makers should consider expanding access to assistance programs and increasing contributions to employer-sponsored resources to further minimize out-of-pocket costs for anticancer medication fills in each benefit year.

Aside from out-of-pocket costs and the use of financial resources,^2^ patient- and provider-level factors may have contributed to patterns of high adherence observed among both HDHP and non-HDHP enrollees. In terms of the former, patients’ awareness of lenalidomide therapy on prolonged survival^16,17^ and improved quality of life^40^ may have motivated them to consistently use medication.^2^ In regard to the latter, high rates (>90%)^41^ of lenalidomide therapy adherence have been associated with frequent health care appointments,^41,42,43^ which provide an opportunity for clinicians to counsel patients on disease severity and the benefits of continuous treatment,^41,42,44^ monitor and address adverse events or adverse effects,^41,44^ and discuss treatment affordability and financial assistance programs.^44^

Last, we observed no significant differences between HDHP enrollment and assignment to either the early or late nonadherence trajectory groups. One possible explanation for our findings is that patients often require multiple lines of therapy throughout the course of multiple myeloma,^17,18^ thus HDHP and non-HDHP enrollees may have been switched to another treatment regimen after lenalidomide therapy intolerance or disease progression.^13,14,16^ Another potential explanation is that HDHP and non-HDHP enrollees may have been prescribed a shorter duration of lenalidomide therapy (eg, 3 cycles of induction therapy)^45^ in preparation for a stem cell transplant—the standard of care among younger adults (younger than 65 years).^46,47^ Given the complexities of multiple myeloma treatment, future research is needed to understand how HDHP enrollment influences the affordability of and access to necessary care throughout the disease course.

### Limitations

This study has several limitations. First, although 40 health plans contribute to the IBM MarketScan database,^48^ our cohort primarily consisted of commercially insured patients employed at large firms,^48^ which limits the generalizability of our findings to those employed by medium or small firms, enrolled in health insurance exchange plans, or covered by public health plans. Second, our analysis focused on a single orally administered anticancer medication. However, the price of lenalidomide therapy is equivalent to other and newly approved anticancer therapies,^49^ and we would expect similar out-of-pocket spending for patients with cancer. Third, the supply of lenalidomide therapy may vary depending on when the medication was prescribed (eg, 28-day supply for maintenance therapy, 21-day supply for initial combination therapy).^50^ Although patients with a shorter supply would be categorized as nonadherent in our study, the treatment-recommended supply should not vary by type of health plan coverage. Fourth, our analysis focused on prescription fills and refills; therefore, we could not determine whether patients actually ingested lenalidomide, nor could we account for medication received while hospitalized. Fifth, although group-based trajectory models have summarized longitudinal adherence better than conventional approaches, they assume that within-person correlation is fully explained by the trajectory curve for each patient’s group, which may not be the case.^27,28,29,30,31,32^ Sixth, our analysis focused on a subset of patients who initiated lenalidomide therapy; thus, we did not observe patients who either delayed treatment or never filled a lenalidomide prescription due to financial burden.^22,51^ As a result, we may have underestimated differences in out-of-pocket spending by enrollment status. Seventh, to ensure adequate sample size for our analyses, we required the cohort to have continuous health plan coverage in the 3 months before treatment initiation. However, some enrollees may have temporarily discontinued and restarted lenalidomide therapy after this 3-month period, which, in turn, may have resulted in different adherence patterns. Eighth, we were not able to identify clinically indicated reasons for nonadherence (eg, therapy nonresponse, adverse event); however, these events should not differ by the generosity of a patient’s health plan. Ninth, we lacked information regarding benefit design (eg, size of deductibles, out-of-pocket limits) and access to and use of health spending accounts and prescription assistance programs. Last, commercial claims lack information related to known contributors to nonadherence, including sociodemographic factors (eg, race and ethnicity, household income), patients’ beliefs and behaviors (eg, understanding of treatment adverse effects), and clinician characteristics (eg, treatment preferences).

## Conclusions

Despite higher observed out-of-pocket costs among commercially insured HDHP enrollees, we did not observe any statistically significant differences in lenalidomide therapy adherence patterns between HDHP and non-HDHP enrollees. Availability of financial resources, frequent contact with the health care system, and patients’ understanding of disease severity and treatment benefits may have contributed to high adherence among enrollees, including those with high upfront out-of-pocket costs. As such, payers, policy makers, and clinicians should consider addressing known barriers to access (eg, out-of-pocket costs, patient education, structural barriers) to ensure consistent use of high-value anticancer medications.

## Notes

Supplement. **eTable 1.** Adjusted Out-of-Pocket Estimates for Initial Lenalidomide Prescription Fill by Spending Quantile **eTable 2.** Adjusted Out-of-Pocket Estimates for Any Lenalidomide Prescription Fill by Spending Quantile **eTable 3.** Group-Based Trajectory Model Fit Criteria **eTable 4.** Association Between HDHP Enrollment and Adherence Group Assignment **eTable 5.** Out-of-Pocket Costs for Initial Lenalidomide Prescription Fill by Spending Quantile and Quarter of Treatment Initiation **eTable 6.** Out-of-Pocket Costs for Any Lenalidomide Prescription Fill by Spending Quantile and Quarter of Treatment Initiation **eTable 7.** Association Between HDHP Enrollment and Paying $0 for Lenalidomide Prescription Fills
