# Supplementary material for: Association of High-Deductible Health Plan Enrollment With Spending on and Use of Lenalidomide Therapy Among Commercially Insured Patients With Multiple Myeloma
Source: JAMA Netw Open. 2022 Jun 7;5(6):e2215720. doi: 10.1001/jamanetworkopen.2022.15720 (PMC9175078; doi:10.1001/jamanetworkopen.2022.15720)
Supplement: Supplement. — eTable 1. Adjusted Out-of-Pocket Estimates for Initial Lenalidomide Prescription Fill by Spending Quantile eTable 2. Adjusted Out-of-Pocket Estimates for Any Lenalidomide Prescription Fill by Spending Quantile eTable 3. Group-Based Trajectory Model Fit Criteria eTable 4. Association Between HDHP Enrollment and Adherence Group Assignment eTable 5. Out-of-Pocket Costs for Initial Lenalidomide Prescription Fill by Spending Quantile and Quarter of Treatment Initiation eTable 6. Out-of-Pocket Costs for Any Lenalidomide Prescription Fill by Spending Quantile and Quarter of Treatment Initiation eTable 7. Association Between HDHP Enrollment and Paying $0 for Lenalidomide Prescription Fills [file jamanetwopen-e2215720-s001.pdf]

Supplementary Online Content

Jazowski SA, Wilson L, Dusetzina SB, Zafar SY, Zullig LL. Association of high-deductible health plan enrollment with spending on and use of lenalidomide therapy among commercially insured patients with multiple myeloma. *JAMA Netw Open*. 2022;5(6):e2215720. doi:10.1001/jamanetworkopen.2022.15720

eTable 1. Adjusted Out-of-Pocket Estimates for Initial Lenalidomide Prescription Fill by Spending Quantile
eTable 2. Adjusted Out-of-Pocket Estimates for Any Lenalidomide Prescription Fill by Spending Quantile
eTable 3. Group-Based Trajectory Model Fit Criteria
eTable 4. Association Between HDHP Enrollment and Adherence Group Assignment
eTable 5. Out-of-Pocket Costs for Initial Lenalidomide Prescription Fill by Spending Quantile and Quarter of Treatment Initiation
eTable 6. Out-of-Pocket Costs for Any Lenalidomide Prescription Fill by Spending Quantile and Quarter of Treatment Initiation
eTable 7. Association Between HDHP Enrollment and Paying $0 for Lenalidomide Prescription Fills

This supplementary material has been provided by the authors to give readers additional information about their work.

© 2022 Jazowski SA et al. *JAMA Network Open*.

eTable 1. Adjusted Out-of-Pocket Estimates for Initial Lenalidomide Prescription Fill by Spending Quantile[a]

	**25th Percentile (95% CI)**	**50th Percentile (95% CI)**	**75th Percentile (95% CI)**	**90th Percentile (95% CI)**	**95th Percentile (95% CI)**
Intercept	-$10.78 (-$28.13-$6.58)	**$53.08** ($20.95-$85.20)	**$120.16** ($34.54-$205.78)	**$1,248.72** ($816.56-$1,680.88)	$1,108.20 (-$588.12-$2,804.51)
HDHP enrollment (ref=HDHP)					
Non-HDHP enrollee	**$13.69** ($9.55-$17.81)	**-$19.03** (-$26.67--$11.38)	**-$38.82** (-$59.2--$18.45)	**-$756.41** (-$859.25--$653.57)	-$376.17 (-$779.83-$27.50)
Age	**$0.21** ($0.01-$0.41)	-$0.06 (-$0.42-$0.30)	$0.60 (-$0.90-$1.02)	-$1.92 (-$6.78-$2.94)	$6.05 (-$13.02-$25.13)
Sex (ref=Women)					
Men	-$2.21 (-$4.73-$0.31)	-$3.08 (-$7.75-$1.59)	-$2.06 (-$14.51-$10.39)	$14.01 (-$48.82-$76.83)	-$2.92 (-$249.52-$243.69)
Region (ref=Unknown)					
Northeast	**-$15.68** (-$26.30--$5.05)	-$7.02 (-$26.68-$12.64)	$3.86 (-$48.54-$56.26)	$13.19 (-$251.29-$277.67)	$117.34 (-$920.79-$1,155.48)
North Central	$3.64 (-$6.98-$14.25)	**$24.29** ($4.63-$43.94)	**$66.48** ($14.09-$118.87)	**$311.84** ($47.41-$576.26)	$914.69 (-$123.23-$1,952.60)
South	**$15.91** ($5.46-$26.36)	**$41.53** ($22.20-$60.86)	**$80.03** ($28.50-$131.56)	$165.65 (-$94.41-$425.78)	$672.86 (-$348.05-$1,693.78)
West	-$2.21 (-$12.99-$8.58)	$14.75 (-$5.22-$34.72)	**$57.10** ($3.87-$110.33)	$163.41 (-$105.25-$432.07)	**$1,059.51** ($4.96-$2,114.06)
Year of initiation (ref=2017)					
2013	**$8.35** ($3.31-$13.83)	$5.37 (-$3.95-$14.68)	**-$27.13** (-$51.95--$2.30)	**-$238.15** (-$363.45--$112.86)	**-$586.56** (-$1078.37--$94.76)
2014	$1.03 (-$3.68-$5.73)	-$5.47 (-$14.18-$3.23)	**-$31.59** (-$54.80--$8.39)	**-$243.51** (-$360.64--$126.37)	**-$694.47** (-$1154.24--$234.69)
2015	-$0.65 (-$5.44-$4.14)	-$0.90 (-$9.76-$7.96)	**-$33.60** (-$57.22--$9.99)	-$110.06 (-$229.24-$9.13)	-$44.79 (-$512.62-$423.03)
2016	-$2.21 (-$6.91-$2.49)	-$8.50 (-$17.21-$0.21)	**-$35.01** (-$58.23--$11.80)	**-$236.09** (-$353.26--$118.92)	**$616.97** (-$1,076.90--$157.03)
Quarter of initiation (ref=Q4)					
Q1	**$14.45** ($10.46-$18.41)	**$23.23** ($15.86-$30.60)	**$63.43** ($43.78-$83.08)	**$1,347.54** ($1,248.36-$1,446.72)	**$2,104.98** ($1,715.68-$2,494.28)
Q2	**$5.00** ($1.37-$8.62)	$4.81 (-$1.91-$11.52)	$12.21 (-$5.69-$30.10)	$40.05 (-$50.28-$130.37)	$308.27 (-$46.27-$662.82)
Q3	**$6.42** ($2.42-$10.41)	$5.87 (-$1.52-$13.26)	$4.67 (-$15.03-$24.38)	$20.35 (-$79.10-$119.80)	$23.84 (-$366.53-$414.20)

© 2022 Jazowski SA et al. *JAMA Network Open*.

Comorbidities (ref=2)					
0	-$1.04<br>(-$5.31-$3.22)	-$3.24<br>(-$11.14-$4.66)	-$3.00<br>(-$24.06-$18.06)	-$40.69<br>(-$146.98-$65.61)	-$96.65<br>(-$513.88-$320.58)
1	-$0.34<br>(-$5.12-$4.44)	-$3.01<br>(-$11.85-$5.84)	-$4.38<br>(-$27.96-$19.21)	-$19.65<br>(-$138.67-$99.37)	-$84.16<br>(-$551.33-$383.01)
Unique medications	**-$0.56**<br>(-$0.85--$0.26)	**-$1.20**<br>(-$1.74--$0.66)	**-$2.34**<br>(-$3.79--$0.89)	**-$7.84**<br>(-$15.15--$0.54)	**-$30.06**<br>(-$58.74--$1.39)

Abbreviation: HDHP, high-deductible health plan

[a] Bolded estimates are statistically significant at $p<0.05$ level.

© 2022 Jazowski SA et al. *JAMA Network Open*.

eTable 2. Adjusted Out-of-Pocket Estimates for Any Lenalidomide Prescription Fill by Spending Quantile[a]

	25th Percentile (95% CI)	50th Percentile (95% CI)	75th Percentile (95% CI)	90th Percentile (95% CI)	95th Percentile (95% CI)
Intercept	**-$6.57** (-$12.23--$0.89)	**$30.98** ($20.67-$41.17)	**$66.66** ($43.51-$89.92)	**$119.56** ($35.12-$204.35)	**$929.43** ($476.44-$1,379.96)
HDHP enrollment (ref=HDHP)					
Non-HDHP enrollee	**$7.57** ($6.19-$8.92)	$1.42 (-$1.00-$3.93)	**-$9.09** (-$14.66--$3.50)	$8.32 (-$11.94-$28.74)	**-$216.72** (-$323.43--$106.28)
Age	**$0.28** ($0.21-$0.34)	**-$0.17** (-$0.28--$0.05)	-$0.04 (-$0.31-$0.22)	-$0.42 (-$1.39-$0.55)	-$0.44 (-$5.63-$4.74)
Sex (ref=Women)					
Men	-$0.83 (-$1.66-$0.00)	**-$4.24** (-$5.73--$2.72)	$0.01 (-$3.43-$3.37)	$9.42 (-$2.96-$21.83)	$27.52 (-$38.62-$93.74)
Region (ref=Unknown)					
Northeast	**-$18.58** (-$21.91--$15.18)	**-$11.35** (-$17.46--$5.30)	$0.41 (-$13.37-$14.15)	$35.27 (-$15.01-$85.35)	$81.77 (-$186.70-$349.14)
North Central	**-$7.46** (-$10.81--$4.08)	**$16.83** ($10.70-$22.86)	**$45.03** ($31.21-$58.74)	**$147.73** ($97.32-$197.72)	**$430.24** ($161.54-$697.55)
South	**-$6.44** (-$9.73--$3.13)	**$28.91** ($22.90-$34.83)	**$54.99** ($41.41-$68.41)	**$128.93** ($79.44-$177.94)	**$276.20** ($12.63-$538.51)
West	**-$10.36** (-$13.76--$6.93)	$5.15 (-$1.04-$11.29)	**$31.03** ($17.01-$44.92)	**$103.77** ($52.63-$154.4)	$240.90 (-$31.15-$512.20)
Year of initiation (ref=2017)					
2013	**$17.21** ($15.52-$18.85)	**$17.94** ($14.88-$20.90)	**$13.89** ($6.95-$20.57)	$1.00 (-$24.24-$25.46)	$26.99 (-$106.31-$159.01)
2014	**$9.14** ($7.56-$10.68)	**$10.43** ($7.56-$13.19)	**$9.70** ($3.19-$15.94)	$6.88 (-$16.73-$29.78)	$26.15 (-$98.52-$149.76)
2015	$1.34 (-$0.25-$2.92)	**$4.93** ($2.04-$7.76)	$5.44 (-$1.14-$11.82)	$11.03 (-$12.89-$34.37)	**$184.22** ($57.52-$309.81)
2016	$1.11 (-$0.45-$2.66)	-$0.31 (-$3.10-$2.51)	-$1.05 (-$7.41-$5.29)	$3.91 (-$19.28-$27.04)	-$17.69 (-$141.71-$105.64)
Quarter of initiation (ref=Q4)					
Q1	**$4.89** ($3.57-$6.20)	**$4.57** ($2.19-$6.95)	$3.77 (-$1.57-$9.20)	$8.97 (-$10.67-$28.63)	**-$114.62** (-$218.40--$8.61)
Q2	-$0.51 (-$1.71-$0.70)	**-$3.28** (-$5.47--$1.12)	**-$14.85** (-$19.72--$9.89)	**-$43.55** (-$61.42--$25.57)	**-$477.43** (-$572.65--$381.24)
Q3	**$1.38** ($0.06-$2.70)	-$2.10 (-$4.48-$0.28)	**-$10.20** (-$15.57--$4.80)	**-$45.91** (-$65.56--$26.27)	**-$446.79** (-$551.20--$341.43)
Comorbidities (ref=2)					
0	**-$2.40** (-$3.84--$0.95)	-$2.19 (-$4.80-$0.41)	-$2.40 (-$8.28-$3.51)	$9.99 (-$11.47-$31.53)	**-$134.43** (-$249.50--$19.95)
1	**-$2.81** (-$4.42$1.20)	-$2.12 (-$5.02-$0.78)	-$0.80 (-$7.35-$5.80)	$3.54 (-$20.36-$27.58)	**-$163.00** (-$290.79--$34.88)

© 2022 Jazowski SA et al. *JAMA Network Open*.

Unique medications	**-$0.32** (-$0.42--$0.22)	**-$0.49** (-$0.67--$0.31)	**-$1.24** (-$1.64--$0.83)	**-$2.53** (-$3.99--$1.05)	-$7.87 (-$15.75--$0.01)

Abbreviation: HDHP, high-deductible health plan

[a] Bolded estimates are statistically significant at p<0.05 level.

© 2022 Jazowski SA et al. *JAMA Network Open*.

eTable 3. Group-Based Trajectory Model Fit Criteria[a]

Number of Groups	BIC	Smallest Group Percentage	Optimal Model
2	-10528.94	45.00	
3	-10347.05	28.25	X
4	-10324.53	16.75	
5	-10327.20	2.95	

Abbreviations: BIC, bayesian information criteria

[a] Assessing overall model fit and selecting the appropriate number of adherence groups (two to five were modeled) was based on the following factors: bayesian information criteria (BIC); estimated group proportions; and power for the multinomial logistic regression model. First, BIC improved from modeling two to three groups and remained stable when modeling four and five groups. Second, since the model with five groups had less than 5% of the population assigned to a group, we compared the models with three and four groups. Last, to maximize power and the interpretability of the multinomial logistic regression model (used to assess the association between high-deductible health plan enrollment and group assignment), we selected the model with 3 adherence groups.

© 2022 Jazowski SA et al. *JAMA Network Open*.

eTable 4. Association Between HDHP Enrollment and Adherence Group Assignment

	Odds Ratio (95% CI)	
	**Unadjusted**	**Adjusted[a]**
Early nonadherers vs. high adherers	1.10 (0.82-1.48)	1.18 (0.87-1.59)
Late nonadherers vs. high adherers	1.25 (0.96-1.63)	1.21 (0.93-1.59)

Abbreviation: HDHP, high-deductible health plan

[a] Model was adjusted for age at lenalidomide therapy initiation, sex, region, quarter and year of lenalidomide therapy initiation, comorbidities, and unique prescriptions at baseline.

© 2022 Jazowski SA et al. *JAMA Network Open*.

eTable 5. Out-of-Pocket Costs for Initial Lenalidomide Prescription Fill by Spending Quantile and Quarter of Treatment Initiation

		HDHP Enrollees		Non-HDHP Enrollees	
		**Unadjusted (95% CI)**	**Adjusted[a] (95% CI)**	**Unadjusted (95% CI)**	**Adjusted[a] (95% CI)**
25th Percentile	Quarter 1	$33.56 ($15.31-$51.81)	$33.19 ($4.76-$61.61)	$27.16 (-$10.24-$64.55)	$19.75 (-$29.31-$68.80)
	Quarter 2	$0.00 (-$17.91-$17.91)	-$4.36 (-$32.37-$23.64)	$10.00 (-$26.69-$46.69)	$11.73 (-$36.51-$59.96)
	Quarter 3	$0.00 (-$18.78-$18.78)	-$6.76 (-$35.69-$22.17)	$16.29 (-$22.21-$54.81)	$13.32 (-$36.86-$63.51)
	Quarter 4	$0.00 (-$8.09-$8.09)	-$11.48 (-$28.89-$5.93)	$0.00 (-$16.56-$16.56)	$7.38 (-$19.17-$33.92)
50th Percentile	Quarter 1	$105.90 ($63.90-$147.88)	$90.94 ($32.64-$149.23)	$56.48 (-$29.59-$142.54)	$57.76 (-$42.86-$158.38)
	Quarter 2	$72.42 ($31.20-$113.63)	$58.20 ($0.75-$115.65)	$44.76 (-$39.68-$129.19)	$41.04 (-$57.91-$139.98)
	Quarter 3	$54.31 ($11.08-$97.54)	$50.79 (-$8.55-$110.13)	$44.76 (-$43.86-$133.38)	$43.59 (-$59.34-$146.53)
	Quarter 4	$50.88 ($32.26-$69.49)	$44.50 ($8.78-$80.22)	$39.95 ($1.83-$78.05)	$36.10 (-$18.35-$90.55)
75th Percentile	Quarter 1	$498.72 ($395.56-$601.87)	$465.19 ($308.37-$622.00)	$158.84 (-$52.56-$370.25)	$139.61 (-$131.08-$410.30)
	Quarter 2	$149.66 ($48.40-$250.90)	$138.89 (-$15.65-$293.43)	$105.90 (-$101.51-$313.30)	$96.13 (-$170.04-$362.30)
	Quarter 3	$111.90 ($5.72-$218.07)	$88.88 (-$70.77-$248.52)	$101.76 (-$115.93-$319.45)	$95.48 (-$181.45-$372.40)
	Quarter 4	$144.84 ($99.10-$190.57)	$130.76 ($34.66-$226.86)	$97.53 ($3.91-$191.14)	$84.18 (-$62.31-$230.66)
90th Percentile	Quarter 1	$3,173.81 ($2,414.45-$3,933.16)	$3,197.59 ($2,483.00-$3,912.17)	$1,553.13 (-$3.15-$3,109.41)	$1,697.24 ($463.74-$2,930.75)
	Quarter 2	$305.28 (-$440.04-$1,050.61)	$547.23 (-$157.01-$1,251.47)	$279.75 (-$1,247.12-$1,806.62)	$543.22 (-$669.70-$1,756.14)
	Quarter 3	$152.64 (-$628.97-$934.26)	$483.37 (-$244.12-$1,210.87)	$223.80 (-$1,378.74-$1,826.34)	$513.50 (-$748.42-$1,775.41)
	Quarter 4	$271.36 (-$65.31-$608.04)	$565.55 ($127.62-$1,003.46)	$217.26 (-$471.89-$906.41)	$496.52 (-$171.03-$1,164.07)
95th Percentile	Quarter 1	$3,846.27 ($556.07-$7,136.47)	$3,368.48 ($770.13-$5,966.81)	$3,103.71 (-$3,639.47-$9,846.89)	$2,849.23 (-$1,635.95-$7,334.41)
	Quarter 2	$1,900.82 (-$1,328.60-$5,130.24)	$1,035.21 (-$1,525.50-$3,595.92)	$1,289.11 (-$5,326.65-$7,904.88)	$1,083.99 (-$3,326.35-$5,494.34)
	Quarter 3	$1,811.01 (-$1,575.64-$5,197.66)	$1,651.01 (-$994.26-$4,296.27)	$434.52 (-$6,509.11-$7,378.15)	$731.23 (-$3,857.28-$5,319.74)
	Quarter 4	$1,417.89 (-$40.90-$2,876.68)	$1,394.81 (-$197.52-$2,987.13)	$841.75 (-$2,144.26-$3,827.77)	$713.11 (-$1,714.18-$3,140.40)

Abbreviation: HDHP, high-deductible health plan

[a] Model was adjusted for age at lenalidomide therapy initiation, sex, region, quarter and year of lenalidomide therapy initiation, comorbidities, unique prescriptions at baseline, and the interaction between HDHP enrollment and quarter of initiation.

© 2022 Jazowski SA et al. *JAMA Network Open*.

eTable 6. Out-of-Pocket Costs for Any Lenalidomide Prescription Fill by Spending Quantile and Quarter of Treatment Initiation

		HDHP Enrollees		Non-HDHP Enrollees	
		**Unadjusted (95% CI)**	**Adjusted[a] (95% CI)**	**Unadjusted (95% CI)**	**Adjusted[a] (95% CI)**
25th Percentile	Quarter 1	$0.00 ($0.00-$0.00)	-$0.40 (-$8.64-$7.76)	$0.00 ($0.00-$0.00)	$9.42 (-$4.88-$23.62)
	Quarter 2	$0.00 ($0.00-$0.00)	-$2.75 (-$10.90-$5.30)	$0.00 ($0.00-$0.00)	$2.81 (-$11.27-$16.82)
	Quarter 3	$0.00 ($0.00-$0.00)	-$2.95 (-$11.35-$5.35)	$0.00 ($0.00-$0.00)	$6.94 (-$7.69-$21.26)
	Quarter 4	$0.00 ($0.00-$0.00)	-$1.60 (-$6.64-$3.35)	$0.00 ($0.00-$0.00)	$2.56 (-$5.16-$10.22)
50th Percentile	Quarter 1	$54.31 ($39.35-$69.09)	$51.02 ($30.91-$71.11)	$40.09 ($9.63-$70.51)	$37.59 ($2.69-$72.52)
	Quarter 2	$0.00 (-$14.63-$14.63)	$14.27 (-$5.48-$34.19)	$33.57 ($3.57-$63.45)	$30.90 (-$3.48-$65.31)
	Quarter 3	$27.97 ($12.63-$43.22)	$23.36 ($2.92-$43.85)	$38.02 ($6.62-$69.28)	$31.86 (-$3.84-$67.57)
	Quarter 4	$44.76 ($38.03-$51.33)	$42.42 ($30.20-$54.69)	$42.36 ($28.71-$55.89)	$33.48 ($14.64-$52.34)
75th Percentile	Quarter 1	$112.96 ($76.76-$148.87)	$71.51 ($27.53-$115.16)	$97.77 ($23.96-$171.58)	$57.53 (-$18.77-$133.44)
	Quarter 2	$100.00 ($64.51-$135.48)	$51.50 ($8.15-$94.67)	$76.32 ($3.71-$148.93)	$38.00 (-$37.24-$112.74)
	Quarter 3	$83.92 ($46.68-$120.88)	$34.16 (-$10.88-$78.35)	$83.92 ($7.81-$159.75)	$43.37 (-$34.67-$121.04)
	Quarter 4	$112.96 ($96.69-$128.93)	$94.93 ($68.03-$121.41)	$101.76 ($68.81-$134.71)	$51.74 ($10.41-$92.60)
90th Percentile	Quarter 1	$217.26 ($61.02-$372.79)	$208.46 ($44.68-$371.87)	$211.79 (-$107.55-$530.62)	$135.47 (-$148.81-$419.56)
	Quarter 2	$152.64 (-0.$77-$306.06)	$74.99 (-$86.53-$236.51)	$167.84 (-$146.34-$481.48)	$102.81 (-$177.18-$382.86)
	Quarter 3	$130.36 (-$30.23-$290.50)	$91.25 (-$75.57-$257.64)	$149.20 (-$179.47-$477.37)	$94.84 (-$195.85-$385.56)
	Quarter 4	$268.56 ($198.41-$337.80)	$191.77 ($91.92-$291.24)	$217.26 ($74.43-$359.35)	$134.24 (-$19.32-$287.56)
95th Percentile	Quarter 1	$1,952.54 ($1,245.81-$2,659.37)	$1,522.03 ($702.82-$2,337.96)	$709.68 (-$738.06-$2,155.68)	$450.65 (-$969.23-$1,871.25)
	Quarter 2	$203.52 (-$492.13-$899.18)	$96.30 (-$710.32-$904.08)	$305.28 (-$1,118.13-$1,728.71)	$196.35 (-$1,202.70-$1,596.17)
	Quarter 3	$942.42 ($213.65-$1,668.03)	$836.01 ($3.23-$1,668.46)	$289.68 (-$1,200.00-$1,778.39)	$191.80 (-$1,260.90-$1,644.75)
	Quarter 4	$1,623.22 ($1,304.49-$1,936.52)	$1,468.54 ($968.50-$1,964.63)	$752.63 ($106.67-$1,398.62)	$583.13 (-$184.18$1,349.52)

Abbreviation: HDHP, high-deductible health plan

[a] Model was adjusted for age at lenalidomide therapy initiation, sex, region, quarter and year of lenalidomide therapy initiation, comorbidities, unique prescriptions at baseline, and the interaction between HDHP enrollment and quarter of initiation.

© 2022 Jazowski SA et al. *JAMA Network Open*.

eTable 7. Association Between HDHP Enrollment and Paying $0 for Lenalidomide Prescription Fills

	N (%)		Risk Ratio (95% CI)	
	**HDHP**	**Non-HDHP**	**Unadjusted**	**Adjusted**[a]
**Initial Fill**				
$0 out-of-pocket costs	96 (29.30%)	640 (22.60%)	1.08 (0.85-1.38)	1.25 (1.04-1.50)
**Any Fill**				
$0 out-of-pocket costs	202 (61.50%)	1,069 (37.70%)	1.63 (1.48-1.80)	1.64 (1.48-1.82)

Abbreviation: HDHP, high-deductible health plan
[a] Model was adjusted for age at lenalidomide therapy initiation, sex, region, quarter and year of lenalidomide therapy initiation, comorbidities, and unique prescriptions at baseline.

© 2022 Jazowski SA et al. *JAMA Network Open*.
